# Performance of ultrasonography screening for breast cancer: a systematic review and meta-analysis

**DOI:** 10.1186/s12885-020-06992-1

**Published:** 2020-06-01

**Authors:** Lei Yang, Shengfeng Wang, Liwen Zhang, Chao Sheng, Fengju Song, Ping Wang, Yubei Huang

**Affiliations:** 1 Key laboratory of Carcinogenesis and Translational Research (Ministry of Education/Beijing), Beijing Office for Cancer Prevention and Control, Peking University Cancer Hospital & Institute, Beijing, 100142 China; 2 Department of Epidemiology & Biostatistics, School of Public Health, Peking University, Beijing, 100191 China; 3 Department of Epidemiology and Biostatistics, Key Laboratory of Cancer Prevention and Therapy (Tianjin), Key Laboratory of Breast Cancer Prevention and Therapy (National Ministry of Education), Tianjin Medical University Cancer Institute and Hospital, Tianjin, 300060 China; 4 National Clinical Research Center for Cancer, Tianjin Medical University Cancer Institute and Hospital, Tianjin, 300060 China

**Keywords:** Breast cancer, Screening, Ultrasonography, Mammography, Supplemental ultrasonography

## Abstract

**Background:**

To investigate the performance of primary ultrasound (P-US) screening for breast cancer, and that of supplemental ultrasound (S-US) screening for breast cancer after negative mammography (MAM).

**Methods:**

Electronic databases (PubMed, Scopus, Web of Science, and Embase) were systematically searched to identify relevant studies published between January 2003 and May 2018. Only high-quality or fair-quality studies reporting any of the following performance values for P-US or S-US screening were included: sensitivity, specificity, cancer detected rate (CDR), recall rate (RR), biopsy rate (BR), proportion of invasive cancers among screening-detected cancers (ProIC), and proportion of node-negative cancers among screening-detected invasive cancers (ProNNIC).

**Results:**

Twenty-three studies were included, including 12 studies in which S-US screening was used after negative MAM and 11 joint screening studies in which both primary MAM (P-MAM) and P-US were used. Meta-analyses revealed that S-US screening could detect 96% [95% confidential intervals (CIs): 82 to 99%] of occult breast cancers missed by MAM and identify 93% (95% CIs: 89 to 96%) of healthy women, with a CDR of 3.0/1000 (95% CIs: 1.8/1000 to 4.6/1000), RR of 8.8% (95% CIs: 5.0 to 13.4%), BR of 3.9% (95% CIs: 2.7 to 5.4%), ProIC of 73.9% (95% CIs: 49.0 to 93.7%), and ProNNIC of 70.9% (95% CIs: 46.0 to 91.6%). Compared with P-MAM screening, P-US screening led to the recall of significantly more women with positive screening results [1.5% (95% CIs:0.6 to 2.3%), *P* = 0.001] and detected significantly more invasive cancers [16.3% (95% CIs: 10.6 to 22.1%), *P* < 0.001]. However, there were no significant differences for other performance measures between the two screening methods, including sensitivity, specificity, CDR, BR, and ProNNIC.

**Conclusions:**

Current evidence suggests that S-US screening could detect occult breast cancers missed by MAM. P-US screening has shown to be comparable to P-MAM screening in women with dense breasts in terms of sensitivity, specificity, cancer detection rate, and biopsy rate, but with higher recall rates and higher detection rates for invasive cancers.

## Background

Cancer is a global public health issue in the world. In 2016, an estimated 17.2 million cancer cases and 8.9 million cancer deaths occurred worldwide [1]. For women, both the most commonly occuring cancer and the leading cause of cancer deaths and disability-adjusted life-years (DALYs) was breast cancer (1.7 million incident cases, 535, 000 deaths, and 14.9 million DALYs) [1]. Over the years, the burden of cancer has shifted from more developed countries to less developed countries [2]. Moreover, the burden is expected to grow worldwide due to the aging of the population and the adoption of lifestyle behaviors such as smoking, poor diet, physical inactivity, and reproductive changes (including lower parity and later age at first birth), particularly in less developed countries [3]. Therefore, broad prevention measures, such as cancer screening, are urgently needed to control this increasing burden, especially in less developed countries.

Mammography (MAM) has been used to screen for breast cancer since the 1970s and is now widely available in developed countries. However, in less developed counties, such as China, MAM is not easily accessible due to several barriers, including insufficient MAM equipment, inadequate insurance coverage for MAM, and widely dispersed populations [3]. Moreover, MAM has a low sensitivity in women with dense breasts [4], who could suffer a higher risk of breast cancer than those without dense breasts [5]. Worrisome researches from Denmark and Netherlands showed that nearly one in every three or half of screening-detected breast cancers represents overdiagnosis, respectively [6, 7].

Recent data indicates that supplemental ultrasonography (S-US) screening could detect occult breast cancers missed by MAM, and primary ultrasonography (P-US) screening seems to perform comparably to primary MAM (P-MAM) screening [8–11]. However, systematic reviews of the performances of S-US or P-US screening have been published only in limited studies. Moreover, among broad screening studies in which both P-MAM and P-US were used, researchers just focused on the performance differences between joint screening and P-MAM screening alone. Limited studies investigated the independent performances of P-US screening. Therefore, we conducted this systematic review and meta-analysis to provide a global profile of S-US screening after negative MAM screening or P-US screening for breast cancers.

## Methods

This meta-analysis was reported in line with the preferred reporting items for a systematic review and meta-analysis of diagnostic test accuracy studies: The PRISMA-DTA Statement [12].

### Types of studies and participants

Randomized-controlled trials (RCTs), prospective or retrospective screening cohort studies focusing on the performance of P-US screening for breast cancer or performance of S-US screening for breast cancer after negative MAM were included. The screening performance included the following indicators: sensitivity, specificity, cancer detected rate (CDR), recall rate (RR), biopsy rate (BR), proportions of invasive cancers among screening-detected cancers (ProIC), and proportions of node-negative invasive cancers among screening-detected invasive cancers (ProNNIC). The types of ultrasonography (US) included were hand-held ultrasonography (HHUS) and automated whole breast ultrasonography (ABUS). Diagnostic studies of patients with histopathologically proven breast cancer or women with suspicious finding after initial screening were excluded. Screening studies for second cancers among women previously diagnosed with breast cancer were also excluded.

### Searching strategies

A comprehensive search was conducted according to the Cochrane handbook guidelines. The American College of Radiology (ACR) developed the Breast Imaging Reporting and Data System (BI-RADS) classification for breast ultrasonography examinations starting in 2003 [13]. Electronic databases (PubMed, Scopus, Web of Science, and Embase) were systematically searched to identify relevant studies published in English between January 2003 and May 2018. Five groups of key words were used in the searching strategies: (1) breast neoplasm, breast cancer, breast carcinoma; (2) ultrasound, ultrasonography; (3) screening; (4) supplemental, supplementary, adjunct, adjunctive, combined, joint, primary, single, alone; (5) sensitivity, specificity, detection rate, recall rate, biopsy rate. Reference lists from retrieved articles were also reviewed. Detailed searching strategies are referred to in the supplementary S1.

### Selection of studies

Two authors independently screened the titles and abstracts of all selected articles to confirm their eligibility. All selected articles were analyzed by EndNote software that allows reviewers to manage articles and detect duplicate publications. When two or more articles from the same trial were selected, the article with the larger sample size, longer duration of follow-up, or the latest results was included. Any disagreement on the selection of articles was discussed and arbitrated by a third author. Details of the selection process are provided in the supplementary S2.

### Data extraction

Two authors independently extracted the following data from the qualifying studies: general information (name of first author, year of publication, and country or countries where the study was performed), design of study (sample size, median age, percent of women with dense breasts among the whole population, type of US, screening mode), performance of US, and information for risk assessment of bias (detailed information referred to in the following section). Since there was not a consistent conclusion that dense breast can be regarded as an independent risk factor of breast cancer [5, 14], in order to avoid bringing ‘high risk’ labels to women with dense breasts, we collected information of dense breast as an attribute for average risk women. All data was entered into STATA 14.0 software for analysis. Any disagreements on data extracted were also discussed and arbitrated by the same third author.

### Risk assessment of bias in included studies

Two investigators critically appraised all included studies independently according to the pre-specified criteria, which were adjusted from the USPSTF’s design-specific criteria and the STARD checklist for reporting diagnostic accuracy studies [15, 16]. The adjusted criteria included 7 items: source of population, sample size, inclusion and exclusion criteria, blinding of test, data completeness, BIRADS criteria, and reference standards. Result of each item was classified as high-risk or low-risk. Detailed information of the adjusted risk assessment criteria of bias refered to supplementary S3.

According to the above-mentioned criteria, high-quality studies were defined as those meeting at least six low-risk items for joint screening studies and five low-risk items for S-US screening studies. Fair-quality studies meet four or five low-risk items for joint screening studies and three or four low-risk items for S-US screening studies. Poor quality studies were defined as those meeting less than four low-risk items for joint screening studies and three low-risk items for S-US screening studies. Poor studies were excluded from the review.

### Data synthesis and analysis

All data were extracted with pre-specified uniform tables and recalculated with uniform methods. The corresponding authors were contacted to obtain any missing information from their studies. For those studies in which the number of ‘examinations’ rather than the number of ‘women’ as the denominator to calculate the detection rate of breast cancer, each woman would be followed up several times, and every time she had an examination. Therefore, each woman would have several examinations in these stuides. In this study, if we changed the number of ‘women’ as the denominator to calculate the detection rate for these studies, the results would significantly be overestimated since the number of ‘women’ was significantly less than the number of ‘examinations’. Therefore, in order to follow the analysis protocol in the original studies and avoid potential overestimate in detection rate, we equate each examination with an independent woman. However, equating each examination with an independent woman could bias the estimate because observations within a woman are not ‘independent’ observations.

Cancer detected rate was defined as any cancer detected (including carcinoma in situ and invasive cancer but not high-risk precancerous lesion) among all examinations/participants. The recall rate was calculated as the number of women recalled for further diagnosed examinations divided by the total number of women who participated the screening. If the number of women recalled for any further diagnosed examinations was not available, the number of women with a positive result of index screening modality was used instead. The biopsy rate was calculated as the number of women recalled for pathological examination divided by the total number of women participated the screening.

The variation in different screening performances attributable to heterogeneity was measured as I^2^. If the *P* value for I^2^ was less than 0.1, significant heterogeneity was indicated among included trials and the random-effect model was used to combine screening performances [17]. Otherwise, the fixed-effect model was used if the *P* value for I^2^ was larger than 0.1. To search for sources of heterogeneity and obtain clinically meaningful estimates, subgroup analyses were conducted according to different studies characteristics, such as sample size > 1000 (Yes/No), all women with dense breasts (Yes/No), type of US (HHUS/ABUS), and quality assessment (Yes/No), whenever possible.

The package “midas” was used to combine sensitivity and specificity, to investigate whether there were potential publication biases among included studies, and to plot the summary receiver operating characteristic (SROC) curve with its 95% confidence and prediction contours [18]. The package “metaprop” was used to combine CDR, RR, BR, ProIC, and ProNNIC [19]. In addition, the package “metan” was used to compare the performances between MAM and US [20].

All meta-analyses were conducted with STATA software (version 14.0). All tests were two-sided, and *P* values of less than 0.05 for all meta-analyses indicated statistical significance.

## Results

Supplementary S2 shows a flowchart of the study selection procedure. The electronic searches yielded 1162 potentially relevant studies, of which 23 eligible studies were included in the final review [9–11, 21–40], including 12 studies in which S-US screening was used after negative MAM and 11 joint screening studies in which both P-MAM and P-US were used.

Table 1 shows the baseline characteristics of the 23 studies. Twelve studies were conducted among women with dense breasts. Twenty studies screened women with HHUS. Twelve studies were conducted among general community women or well-defined high-risk women. Eleven studies excluded women who had a personal history of breast cancer. Eight joint screening studies masked the results of P-MAM screening and P-US screening. Nineteen studies had low risk of incomplete data. Sixteen studies reported US results according to BI-RADS classification criteria. The reference standard in seventeen studies was pathologic examination combined with 12-month clinical follow-up. Finally, according to the pre-specified criteria, seven studies were of high quality, while the remaining 16 were of fair quality.

**Table 1:** Characteristics of included studies

Author, year	Country	Age, years	PerDB, %	Type of US	Sample size	Screening mode	Exclusion of BC	Blinding	Complete data	BIRADS criteria	FU, months	Quality assessment	Cohort type
**Supplemental US screening studies**													
Tagliafico, 2016 [21]	Italy [21]	51	100	HHUS	3231	Community	Yes	–	Yes	No	< 12	Fair	Prospective
Kim, 2016 [22]	South Korea	NR	100	HHUS	3171	Opportunistic	Yes	–	Yes	No	12	Fair	Retrospective
Weigert, 2015 [26]	United States	NR	100	HHUS	10,282	Opportunistic	NR	–	Yes	Yes	6	Fair	Retrospective
Hwang, 2015 [25]	South Korea	50	78	HHUS	1727	Opportunistic	No	–	No	Yes	12	Fair	Retrospective
Moon, 2015 [24]	South Korea	53	64	HHUS	2005	Opportunistic	NR	–	Yes	Yes	24	Fair	Retrospective
Parris, 2013 [28]	United States	52	100	HHUS	5519	Opportunistic	No	–	Yes	Yes	NR	Fair	Retrospective
Girardi, 2013 [27]	Italy	51	45	HHUS	22,131	Opportunistic	No	–	Yes	Yes	NR	Fair	Retrospective
Leong, 2012 [32]	Singapore	45	100	HHUS	106	Community	No	–	Yes	No	12–24	Fair	Prospective
Hooley, 2012 [31]	United States	52	100	HHUS	648	Opportunistic	No	–	Yes	Yes	> 15	Fair	Retrospective
Corsetti, 2011 [33]	Italy	NR	100	HHUS	3356	Opportunistic	Yes	–	Yes	No	12	Fair	Retrospective
Youk, 2011 [34]	South Korea	48	100	HHUS	446	Opportunistic	No	–	Yes	Yes	24	Fair	Retrospective
Brancato, 2007 [36]	Italy	52	100	HHUS	5227	Opportunistic	NR	–	Yes	Yes	NR	Fair	Prospective
**Joint screening studies**													
Dong, 2017 [9]	China	52	44	HHUS	31,918	Community	Yes	Yes	Yes	No	12	High	Prospective
Ohuchi, 2016 [10]	Japan	44	NR	HHUS	36,752	Community	Yes	Yes	Yes	Yes	12	High	Prospective
Berg, 2016 [11]	United States	55	100	HHUS	2662	High-risk	Yes	Yes	Yes	Yes	> 12	High	Prospective
Shen, 2015 [23]	China	46	NR	HHUS	4135	High-risk	Yes	Yes	No	Yes	12	High	Prospective
Brem, 2015 [39]	United States	53	100	ABUS	15,318	Community	Yes	No	Yes	Yes	12	High	Prospective
Huang, 2012 [30]	China	46	48	HHUS	3028	Opportunistic	Yes	Yes	Yes	Yes	12	High	Prospective
Kelly, 2010 [40]	United States	53	68	ABUS	4419	High-risk	No	Yes	Yes	Yes	12	High	Prospective
Wilczek, 2016 [38]	Sweden	50	100	ABUS	1668	Community	Yes	No	Yes	No	24	Fair	Prospective
Venturini, 2013 [29]	Italy	46	55	HHUS	1666	Community	Yes	No	No	Yes	6	Fair	Prospective
Weinstein, 2009 [35]	United States	49	60	HHUS	609	High-risk	No	Yes	No	Yes	12	Fair	Prospective
Honjo, 2007 [37]	Japan	NR	NR	HHUS	3453	Community	NR	Yes	Yes	No	≥18	Fair	Prospective

*PerDB* Percent of women with dense breasts accounted for the whole population; *US* Ultrasonography; *BC* Breast cancer; *BIRADS* Breast Imaging-Reporting and Data System; *FU* Follow-up; *HHUS/ABUS* Hand-held / automated breast ultrasonography

### Screening accuracy for S-US and P-US screening

**Fig. 1:**
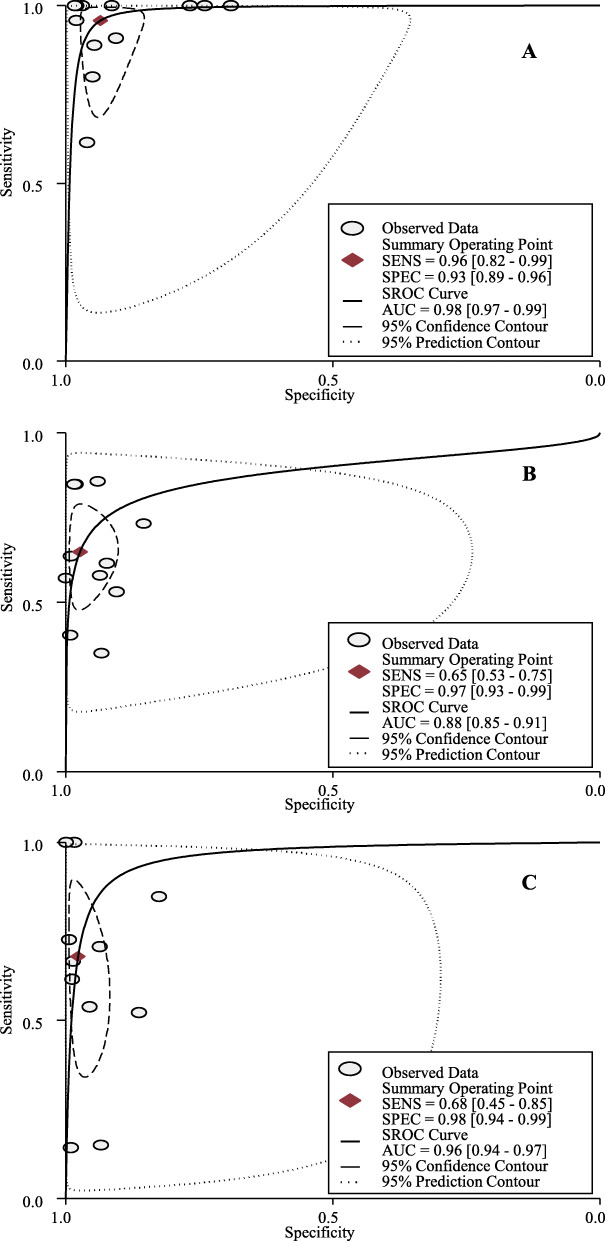
Summary receiver operating characteristic (SROC) curve for S-US screening (**a**), P-MAM screening (**b**), and P-US screening (**c**) for breast cancer

Table 2 shows the original data of screening accuracy for S-US and P-US screening among the included studies. Based on meta-analyses, S-US screening could detect 96% [95% confidential intervals (CIs): 82 to 99%; I^2^ = 64.9%, *P* < 0.01] of occult breast cancers missed by MAM and identify 93% (95% CIs: 89 to 96%; I^2^ = 99.8%, *P* < 0.01) of healthy women (Fig. 1a, supplementary S4). The area under the SROC (AUC) for S-US screening was 98% (95CIs: 97 to 99%) (Fig. 1a). No publication bias was found among these studies (*P* = 0.397).

**Table 2:** Screening accuracy for supplemental and primary US screening

Author, year	Method	Case		Non-case		Sensitivity (95% CI)	Specificity (95% CI)
		+	–	+	–		
**Supplemental US screening studies**							
Tagliafico, 2016 [21]	Supplemental US	23	1	65	3142	0.96(0.77–1.00)	0.98(0.97–0.98)
Kim, 2016 [22]	Supplemental US	9	0	822	2340	1.00(0.63–1.00)	0.74(0.72–0.76)
Weigert, 2015 [26]	Supplemental US	24	15	411	9832	0.62(0.45–0.76)	0.96(0.96–0.96)
Hwang, 2015 [25]	Supplemental US	8	1	92	1626	0.89(0.51–0.99)	0.95(0.93–0.96)
Moon, 2015 [24]	Supplemental US	4	0	619	1382	1.00(0.40–1.00)	0.69(0.67–0.71)
Parris, 2013 [28]	Supplemental US	10	0	171	5338	1.00(0.66–1.00)	0.97(0.96–0.97)
Girardi, 2013 [27]	Supplemental US	41	0	381	21,709	1.00(0.89–1.00)	0.98(0.98–0.98)
Leong, 2012 [32]	Supplemental US	2	0	12	127	1.00(0.20–1.00)	0.91(0.85–0.95)
Hooley, 2012 [31]	Supplemental US	3	0	150	495	1.00(0.31–1.00)	0.77(0.73–0.80)
Corsetti, 2011 [33]	Supplemental US	32	8	363	6821	0.80(0.64–0.90)	0.95(0.94–0.95)
Youk, 2011 [34]	Supplemental US	10	1	41	394	0.91(0.57–1.00)	0.91(0.87–0.93)
Brancato, 2007 [36]	Supplemental US	2	0	106	5119	1.00(0.20–1.00)	0.98(0.98–0.98)
**Joint screening studies**							
Dong, 2017 [9]	Primary MAM	84	15	604	31,215	0.85(0.76–0.91)	0.98(0.98–0.98)
	Primary US	61	38	389	31,430	0.62(0.51–0.71)	0.99(0.99–0.99)
Ohuchi, 2016 [10]	Primary MAM	117	85	2300	33,547	0.58(0.51–0.65)	0.94(0.93–0.94)
	Primary US	143	59	2289	33,558	0.71(0.64–0.77)	0.94(0.93–0.94)
Berg, 2016 [11]	Primary MAM	59	52	700	6662	0.53(0.43–0.63)	0.90(0.90–0.91)
	Primary US	58	53	1012	6350	0.52(0.43–0.62)	0.86(0.85–0.87)
Shen, 2015 [23]	Primary MAM	8	6	3	6913	0.57(0.30–0.81)	1.00(1.00–1.00)
	Primary US	14	0	6	6910	1.00(0.73–1.00)	1.00(1.00–1.00)
Brem, 2015 [39]	Primary MAM	82	30	2219	12,987	0.73(0.64–0.81)	0.85(0.85–0.86)
	Primary US	95	17	2656	12,550	0.85(0.77–0.91)	0.83(0.82–0.83)
Huang, 2012 [30]	Primary MAM	28	5	48	2947	0.85(0.67–0.94)	0.98(0.98–0.99)
	Primary US	24	9	19	2976	0.73(0.54–0.86)	0.99(0.99–1.00)
Kelly, 2010 [40]	Primary MAM	23	34	36	4326	0.40(0.28–0.54)	0.99(0.99–0.99)
	Primary US	38	19	61	4301	0.67(0.53–0.78)	0.99(0.98–0.99)
Wilczek, 2016 [38]	Primary MAM	7	4	16	1641	0.64(0.32–0.88)	0.99(0.98–0.99)
	Primary US	11	0	27	1630	1.00(0.68–1.00)	0.98(0.98–0.99)
Venturini, 2013 [29]	Primary MAM	12	2	99	1553	0.86(0.56–0.97)	0.94(0.93–0.95)
	Primary US	2	12	8	813	0.14(0.03–0.44)	0.99(0.98–1.00)
Weinstein, 2009 [35]	Primary MAM	7	13	37	512	0.35(0.16–0.59)	0.93(0.91–0.95)
	Primary US	3	17	36	511	0.15(0.04–0.39)	0.93(0.91–0.95)
Honjo, 2007 [37]	Primary MAM	8	5	271	3259	0.62(0.32–0.85)	0.92(0.91–0.93)
	Primary US	7	6	158	3372	0.54(0.26–0.80)	0.96(0.95–0.96)

*CI* Confidential interval; *MAM* Mammography; *US* Ultrasonography

**Fig. 2:**
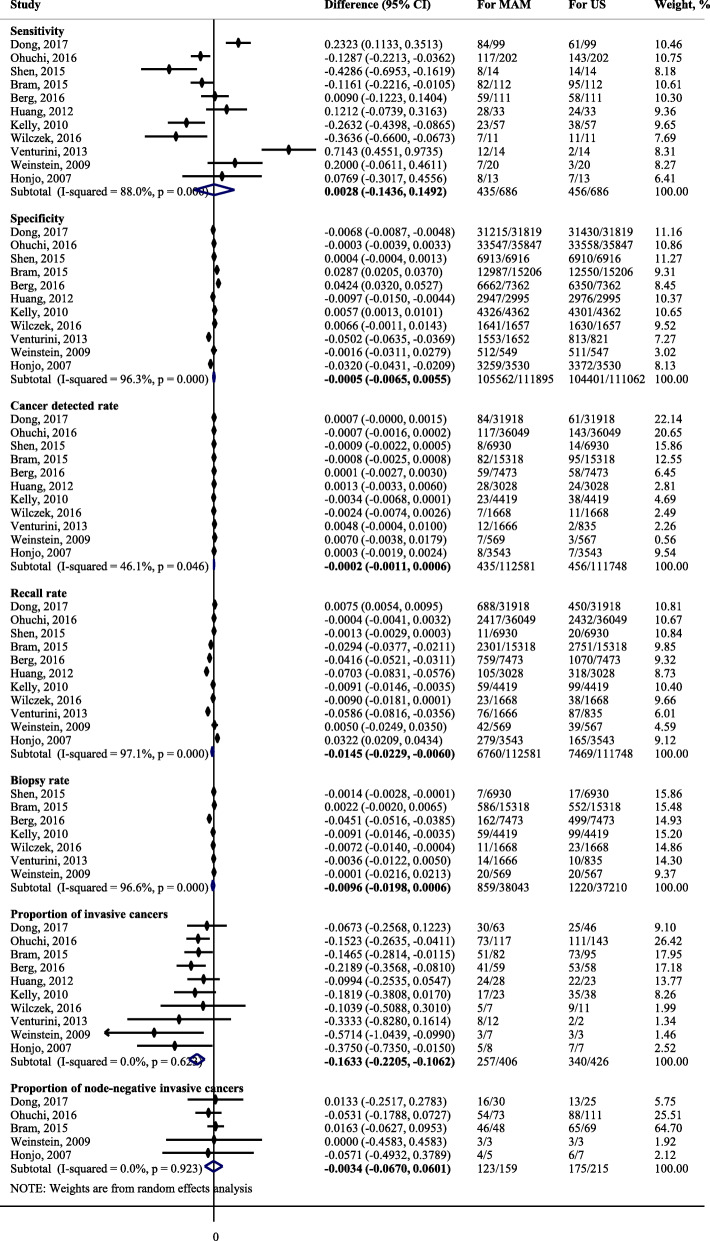
Comparisons on the performances for P-MAM and P-US screening for breast cancer

Among 11 joint screening studies, P-MAM screening could detect 65% (95% CIs: 53 to 75%; I^2^ = 93.2%, *P* < 0.01) of breast cancers and identify 97% (95% CIs: 93 to 99%; I^2^ = 99.9%, *P* < 0.01) of healthy women (Fig. 1b, supplementary S5), respectively. P-US screening could detect 68% (95% CIs: 45 to 85%; I^2^ = 96.2%, *P* < 0.01) of breast cancers and identify 98% (95CIs: 94 to 99%; I^2^ = 100%, *P* < 0.01) of healthy women (Fig. 1c, supplementary S6). The AUCs for P-MAM screening and P-US screening were 88% (95CIs: 85 to 91%) (Fig. 1b) and 96% (95CIs: 94 to 97%) (Fig. 1c), respectively. No publication bias was found for both P-MAM screening (*P* = 0.215) and P-US screening (*P* = 0.266). No significant differences were found for either sensitivity [0.3% (95% CIs: − 14.4 to 14.9%), *P* = 0.970; I^2^ = 88.0%, *P* < 0.001] or specificity [− 0.1% (95% CIs: − 0.7 to 0.5%), *P* = 0.860; I^2^ = 96.3%, *P* < 0.001] between P-MAM screening and P-US screening (Fig. 2).

### Screening efficacy for S-US and P-US screening

**Fig. 3:**
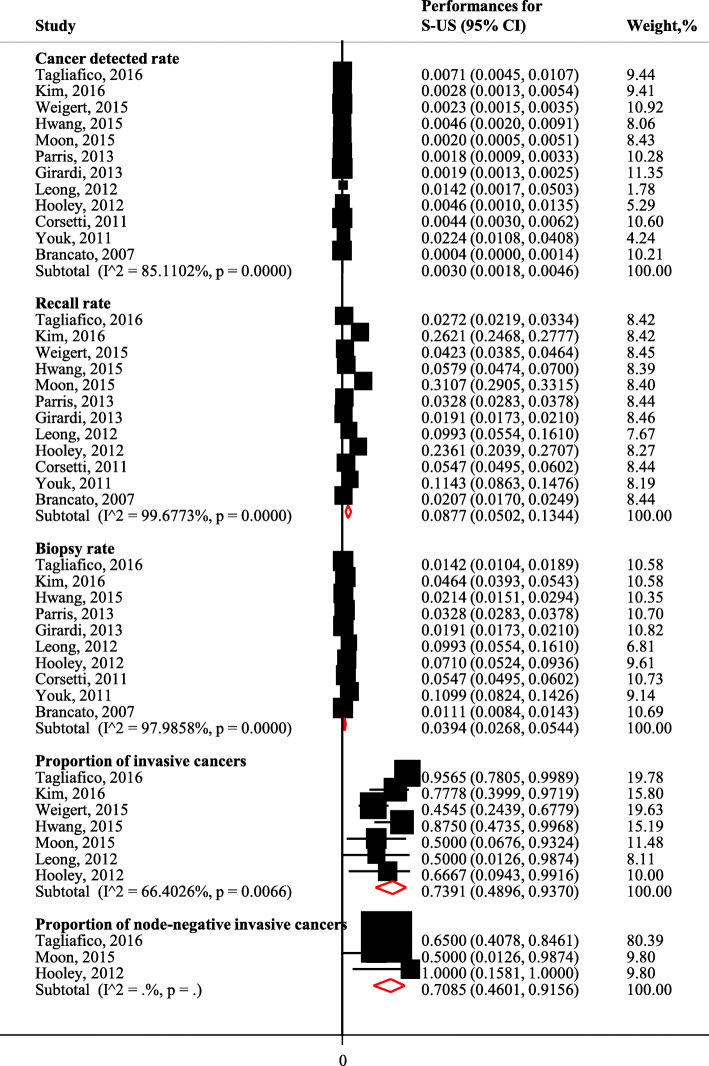
Screening efficacy for S-US screening for breast cancer

Table 3 shows the original data for screening accuracy for S-US and P-US screening reported by the included studies. Meta-analyses showed that the summary CDR for S-US screening was 3.0/1000 (95% CIs: 1.8/1000 to 4.6/1000; I^2^ = 85.1%, *P* < 0.001), with a RR of 8.8% (95% CIs: 5.0 to 13.4%; I^2^ = 99.7%, *P* < 0.001) and a BR of 3.9% (95% CIs: 2.7 to 5.4%; I^2^ = 98.0%, *P* < 0.001) (Fig. 3).

**Table 3:** Screening efficacy for supplemental and primary US screening

Author, year	Method	Cancer detected rate, 1/1000		Recall rate, %		Biopsy rate, %	
		Number	95% CI	Number	95% CI	Number	95% CI
**Supplemental US screening studies**							
Tagliafico, 2016 [21]	Supplemental US	23/3231 women	7.1(4.6–10.8)	88/3231	2.7(2.2–3.4)	46/3231	1.4(1.1–1.9)
Kim, 2016 [22]	Supplemental US	9/3171 women	2.8(1.4–5.6)	831/3171	26.2(24.7–27.8)	147/3171	4.6(3.9–5.4)
Weigert, 2015 [26]	Supplemental US	24/10282 women	2.3(1.5–3.5)	435/10282	4.2(3.9–4.6)		
Hwang, 2015 [25]	Supplemental US	8/1727 women	4.6(2.2–9.5)	100/1727	5.8(4.8–7.0)	37/1727	2.1(1.5–3.0)
Moon, 2015 [24]	Supplemental US	4/2005 women	2.0(0.6–5.5)	623/2005	31.1(29.1–33.2)		
Parris, 2013 [28]	Supplemental US	10/5519 women	1.8(0.9–3.4)	181/5519	3.3(2.8–3.8)	181/5519	3.3(2.8–3.8)
Girardi, 2013 [27]	Supplemental US	41/22131 women	1.9(1.3–2.5)	422/22131	1.9(1.7–2.1)	422/22131	1.9(1.7–2.1)
Leong, 2012 [32]	Supplemental US	2/141 women	14.2(2.5–55.5)	14/141	9.9(5.7–16.4)	14/141	9.9(5.7–16.4)
Hooley, 2012 [31]	Supplemental US	3/648 women	4.6(1.2–14.7)	153/648	23.6(20.4–27.1)	46/648	7.1(5.3–9.4)
Corsetti, 2011 [33]	Supplemental US	32/7224 examinations	4.4(3.1–6.3)	395/7224	5.5(5.0–6.0)	395/7224	5.5(5.0–6.0)
Youk, 2011 [34]	Supplemental US	10/446 examinations	22.4(11.4–42.2)	51/446	11.4(8.7–14.8)	49/446	11.0(8.3–14.4)
Brancato, 2007 [36]	Supplemental US	2/5227 women	0.4(0.1–1.5)	108/5227	2.1(1.7–2.5)	58/5227	1.1(0.9–1.4)
**Joint screening studies**							
Dong, 2017 [9]	Primary MAM	84/31918 women	2.6(2.1–3.3)	688/31918	2.2(2.0–2.3)		
	Primary US	61/31918 women	1.9(1.5–2.5)	450/31918	1.4(1.3–1.5)		
Ohuchi, 2016 [10]	Primary MAM	117/36049 women	3.2(2.7–3.9)	2417/36049	6.7(6.4–7.0)		
	Primary US	143/36049 women	4.0(3.4–4.7)	2432/36049	6.7(6.5–7.0)		
Berg, 2016 [11]	Primary MAM	59/7473 examinations	7.9(6.1–10.2)	759/7473	10.2(9.5–10.9)	162/7473	2.2(1.9–2.5)
	Primary US	58/7473 examinations	7.8(6.0–10.1)	1070/7473	14.3(13.5–15.1)	499/7473	6.7(6.1–7.3)
Shen, 2015 [23]	Primary MAM	8/6930 examinations	1.2(0.5–2.4)	11/6930	0.2(0.1–0.3)	7/6930	0.1(0.0–0.2)
	Primary US	14/6930 examinations	2.0(1.2–3.5)	20/6930	0.3(0.2–0.5)	17/6930	0.2(0.1–0.4)
Brem, 2015 [39]	Primary MAM	82/15318 women	5.4(4.3–6.7)	2301/15318	15.0(14.5–15.6)	586/15318	3.8(3.5–4.1)
	Primary US	95/15318 women	6.2(5.0–7.6)	2751/15318	18.0(17.4–18.6)	552/15318	3.6(3.3–3.9)
Huang, 2012 [30]	Primary MAM	28/3028 women	9.2(6.3–13.5)	105/3028	3.5(2.9–4.2)		
	Primary US	24/3028 women	7.9(5.2–12.0)	318/3028	10.5(9.4–11.7)		
Kelly, 2010 [40]	Primary MAM	23/4419 women	5.2(3.4–7.9)	59/4419	1.3(1.0–1.7)	59/4419	1.3(1.0–1.7)
	Primary US	38/4419 women	8.6(6.2–11.9)	99/4419	2.2(1.8–2.7)	99/4419	2.2(1.8–2.7)
Wilczek, 2016 [38]	Primary MAM	7/1668 women	4.2(1.8–9.0)	23/1668	1.4(0.9–2.1)	11/1668	0.7(0.3–1.2)
	Primary US	11/1668 women	6.6(3.5–12.2)	38/1668	2.3(1.6–3.1)	23/1668	1.4(0.9–2.1)
Venturini, 2013 [29]	Primary MAM	12/1666 women	7.2(3.9–12.9)	76/1666	4.6(3.6–5.7)	14/1666	0.8(0.5–1.4)
	Primary US	2/835 women	2.4(0.4–9.6)	87/835	10.4(8.5–12.7)	10/835	1.2(0.6–2.3)
Weinstein, 2009 [35]	Primary MAM	7/569 women	12.3(5.4–26.3)	42/569	6.9(5.1–9.3)	20/569	3.3(2.1–5.1)
	Primary US	3/567 women	5.3(1.4–16.7)	39/567	6.9(5.0–9.4)	20/567	3.5(2.2–5.5)
Honjo, 2007 [37]	Primary MAM	8/3543 women	2.3(1.1–4.6)	279/3543	7.9(7.0–8.8)		
	Primary US	5/3543 women	2.0(0.9–4.3)	165/3543	4.7(4.0–5.4)		

*CI* Confidential interval**;***MAM* Mammography; *US* Ultrasonography

**Fig. 4:**
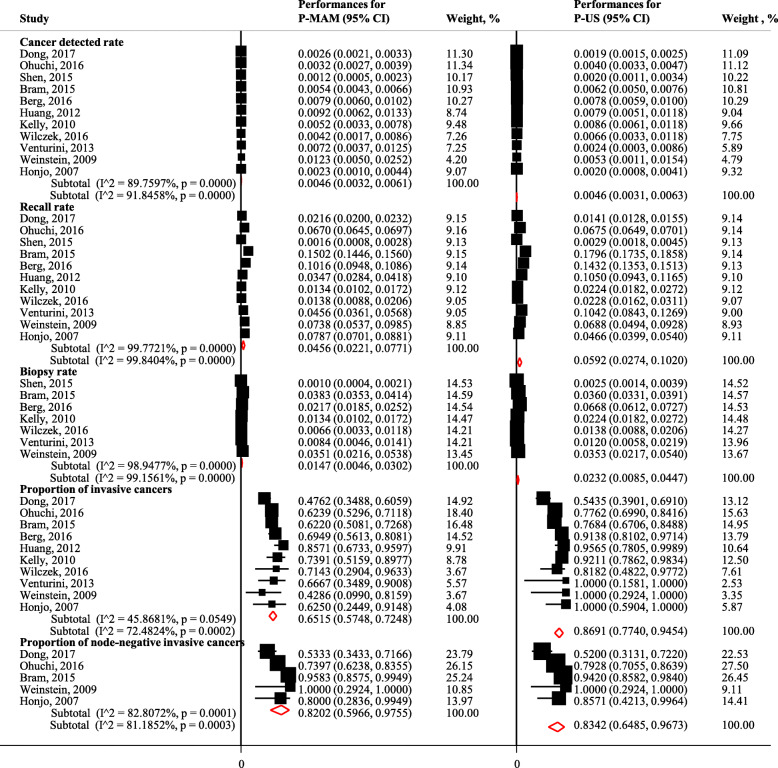
Screening efficacy for P-MAM and P-US screening for breast cancer

Among 11 joint screening studies, the summary CDRs for P-MAM screening and P-US screening were 4.6/1000 (95% CIs: 3.2/1000 to 6.1/1000; I^2^ = 89.8%, *P* < 0.001) and 4.6/1000 (95% CIs: 3.1/1000 to 6.3/1000; I^2^ = 91.9%, *P* < 0.001), with summary RRs of 4.6% (95% CIs: 2.2 to 7.7%; I^2^ = 99.8%, *P* < 0.001) and 5.9% (95% CIs: 2.7 to 10.2%; I^2^ = 99.8%, *P* < 0.001), and summary BRs of 1.5% (95% CIs: 0.5 to 3.0%; I^2^ = 98.9%, *P* < 0.001) and 2.3% (95% CIs: 0.9 to 4.5%; I^2^ = 99.2%, *P* < 0.001) (Fig. 4). Compared to P-MAM screening, P-US screening recalled significantly more women with positive screening results [1.5% (95% CIs: 0.6 to 2.3%), *P* = 0.001] (Fig. 2). No significant differences were found for either CDR [− 0.2/1000 (95% CIs:-1.1/1000 to 0.6/1000, *P* = 0.581; I^2^ = 46.1%, *P* = 0.046] or BR [− 1.0% (95% CIs: − 2.0 to 0.6%), *P* = 0.066; I^2^ = 96.6%, *P* < 0.001] for P-MAM screening compared to P-US screening (Fig. 2).

### Cancer characteristics for S-US and P-US screening

Table 4 shows the original data for cancer characteristics for S-US and P-US screening reported by the included studies. The studies from Corsetti [13], Hwang [25], Youk [32], and Brancato [34] among the S-US screening studies, as well as Shen [21] among joint screening studies did not report detailed information of invasive cancers or node-negative invasive cancers among screening detected cancers, therefore, they are missed in Table 4. After meta-analyses, 73.9% (95% CIs: 49.0 to 93.7%; I^2^ = 66.4%, *P* = 0.007) of cancers detected by S-US screening were invasive cancers, while 70.9% (95% CIs: 46.0 to 91.6%) of cancers were node-negative invasive cancers (Fig. 3).

**Table 4:** Cancer characteristics for supplemental and primary US screening for breast cancer

Author, year	Method	Proportions of invasive cancers, %		Proportions of node-negative invasive cancers, %	
		Number	95% CI	Number	95% CI
**Supplemental US screening studies**					
Tagliafico, 2016 [21]	Supplemental US	22/23	95.7(76.0–99.8)	13/20	65.0(40.9–83.7)
Kim, 2016 [22]	Supplemental US	7/9	77.8(40.2–96.1)		
Weigert, 2015 [26]	Supplemental US	10/22	45.5(25.1–67.3)		
Hwang, 2015 [25]	Supplemental US	7/8	87.5(46.7–99.3)		
Moon, 2015 [24]	Supplemental US	2/4	50.0(15.0–85.0)	1/2	50.0(9.5–90.5)
Leong, 2012 [32]	Supplemental US	1/2	50.0(9.5–90.5)		
Hooley, 2012 [31]	Supplemental US	2/3	66.7(12.5–98.2)	2/2	100.0(19.8–100.0)
**Joint screening studies**					
Dong, 2017 [9]	Primary MAM	30/63	47.6(35.0–60.5)	16/30	53.3(34.6–71.2)
	Primary US	25/46	54.3(39.2–68.8)	13/25	52.0(31.8–71.7)
Ohuchi, 2016 [10]	Primary MAM	73/117	62.4(52.9–71.0)	54/73	74.0(62.2–83.2)
	Primary US	111/143	77.6(69.7–84.0)	89/111	79.3(70.3–86.2)
Berg, 2016 [11]	Primary MAM	41/59	69.5(56.0–80.5)		
	Primary US	53/58	91.4(80.3–96.8)		
Brem, 2015 [39]	Primary MAM	51/82	62.2(50.8–72.5)	46/48	95.8(84.6–99.3)
	Primary US	73/95	76.8(66.8–84.6)	65/69	94.2(85.1–98.1)
Huang, 2012 [30]	Primary MAM	24/28	85.7(66.4–95.3)		
	Primary US	22/23	95.7(76.0–99.8)		
Kelly, 2010 [40]	Primary MAM	17/23	73.9(51.3–88.9)		
	Primary US	35/38	92.1(77.5–97.9)		
Wilczek, 2016 [38]	Primary MAM	5/7	71.4(30.3–94.9)		
	Primary US	9/11	81.8(47.8–96.8)		
Venturini, 2013 [29]	Primary MAM	8/12	66.7(35.4–88.7)		
	Primary US	2/2	100.0(19.8–100.0)		
Weinstein, 2009 [35]	Primary MAM	3/7	42.9(11.8–79.8)	3/3	100.0(31.0–100.0)
	Primary US	3/3	100.0(31.0–100.0)	3/3	100.0(31.0–100.0)
Honjo, 2007 [37]	Primary MAM	5/8	62.5(25.9–89.8)	4/5	80.0(29.9–98.9)
	Primary US	7/7	100.0(56.1–100.0)	6/7	85.7(42.0–99.2)

*CI* Confidential interval**;***MAM* Mammography; *US* Ultrasonography

Among 11 joint screening studies, 65.1% (95% CIs: 57.5 to 72.5%; I^2^ = 45.9%, *P =* 0.055) and 86.9% (95% CIs: 77.4 to 94.5%; I^2^ = 72.5%, *P* < 0.001) of cancers detected by P-MAM screening and by P-US were invasive cancers, while 82.0% (95% CIs: 59.7 to 97.6%; I^2^ = 82.8%, *P* < 0.001) and 83.4% (95% CIs: 64.9 to 96.7%; I^2^ = 81.2%, *P* < 0.001) of cancers were node-negative invasive cancers (Fig. 4). Compared to P-MAM screening, P-US screening detected significantly more invasive cancers [16.3, 95% CIs (10.6 to 22.1%), *P* < 0.001; I^2^ = 0, *P* = 0.623] but a similar number of node-negative invasive cancers [0.3, 95% CIs (− 6.0 to 6.7%), *P* = 0.916; I^2^ = 0, *P* = 0.923] (Fig. 2).

### Subgroup analyses

Subgroup analyses showed very similar results to those of primary analyses (Supplementary S7 and S8). In addition to results comparable to those observed in the primary analyses, lower sensitivity, higher specificity, higher cancer detection rate, and higher biopsy rate were found for S-US screening among women with dense breasts compared to those without dense breasts (Supplementary S7). Moreover, the differences for sensitivities, specificities, and cancer detection rates between P-MAM screening and P-US screening were larger among women with dense breasts compared to those without dense breasts (Supplementary S8).

## Discussion

The U.S. Preventive Services Task Force (USPSTF) had initially reviewed the performances and clinical outcomes of S-US screening in women with dense breasts or negative mammography [15]. However, only two studies were included. The authors concluded that the effects of S-US screening on breast cancer outcomes remain unclear due to sparse good evidence [15]. In addition, Gartlehnerhad systematically reviewed the evidence investigating the joint effectiveness of screening with P-MAM and P-US compared to MAM screening alone [41]. However, this review did not investigate the performance of P-US screening. Our study is the first systematic review and meta-analysis to investigate the performance of P-US screening for breast cancer, and this is also an important up-to-date systematic review and meta-analysis investigating the performance of S-US screening.

The role of S-US screening was first addressed in ACRIN 6666 by Berg in 2008 [4]. Berg concluded that S-US screening to P-MAM screening would yield an additional 1.1 to 7.2 cancers per 1000 high-risk women [4]. Our analyses also found a similar additional 0.4 to 22.4 cancers per 1000 examinations. Moreover, after re-analysis of ACRIN 6666, Berg concluded that ultrasound could be used as the primary screening method for breast cancer [11]. However, up to now, there have been no consistent conclusions concerning whether US screening should be recommended as the primary screening method for women in the screening guidelines for breast cancer. For example, the National Comprehensive Cancer Network, the European Society of Breast Imaging (EUSOBI), the Japanese Breast Cancer Society, and the Chinese Anti-Cancer Association (CACA) supported that S-US screening should be recommended for women with dense breasts after negative MAM [42–45], while no clear recommendations of US screening were suggested by the USPSTF, the American Cancer Society, the American College of Physicians, and the Canadian Task Force on Preventive Health Care [46–49].

Several reasons would lead to these inconsistent recommendations among current guidelines. As argued by USPSTF, sparse good evidence would be the major reason. However, as shown in our study, several high-quality studies and fair-quality studies had been conducted since 2003. Although EUSOBI supported S-US screening after P-MAM, it also addressed the concern that breast US was inappropriately suggested to be a primary screening method since P-US screening had not been shown to reduce mortality of breast cancer in the general female population. Moreover, US would lead to more biopsies and recalls than MAM [45]. In this systematic review, we did observe higher recall rates for US compared to MAM. We also observed higher biopsy rates for US compared to MAM; however, the difference was nonsignificant. This nonsignificant difference in biopsy rates between US and MAM may be due to small sample sizes, but it may also reflect no actual difference. In addition, there are several limitations of breast ultrasound that would make it inappropriate for a screening test. These included: US cannot take an image of the whole breast at once as MAM does; US cannot show microcalcifications, which would be the most common feature of tissue around a tumor; the skill level of the US operators makes a great difference in the screening results. However, as shown in our study, these concerns seemed not to cause significant differences in the sensitivity and specificity, or even in cancer detection rates and cancer characteristics (such as the proportion of node-negative invasive cancers) between P-US screening and P-MAM screening. Moreover, lower price, larger coverage, absence of radiation effects, and lower overdiagnosis rates for US compared to MAM make US more easily accepted in China and other countries [3, 9, 50].Therefore, Chinese Anti-Cancer Association and other societies supported S-US screening in their guidelines.

Lastly, the following results are significant. First, we observed significantly higher RR and ProIC for P-US screening compared with P-MAM screening. Higher recall rates would be an important barrier to promote US screening. More studies are needed to investigate the factors associated with higher recall rates of US screening to reduce unnecessary recalls. Second, as shown in supplementary S7, subgroup analyses did not find obvious differences in sensitivity, specificity or cancer detection rate for S-US screening after negative MAM screening between women with and without dense breasts. These results suggested that influence of dense breasts on the performance of S-US after negative MAM would be influenced by other factors. Moreover, as shown in supplementary S8, subgroup analyses also did not find significantly higher sensitivity for P-MAM compared to P-US among women with dense breasts. Small sample size could be an important factor, since only 3/11 exclusively recruited women with dense breasts (a proportion of 100% dense breasts) and only > 40% of participating women had dense breasts in another 5/11 studies.

### Limitations

First, due to lack of evidence for reduced mortality from breast cancer, we cannot conclude that US screening would lead to a long-term benefit. More studies with sophistacted design and long-time follow-up are needed to investigate the long-term benefits and potential risks (including false positivity, “unnecessary” recalls, and overdiagnosis) of P-US screening. Second, in addition to breast density, no studies investigated whether other risk factors (such as obesity) influenced the differences in screening performance between US and MAM. Therefore, we cannot conclude whether these different performances between US and MAM derived from confounding effects or from the actual differences between US and MAM. Third, as shown in Table 3 and Table 4, not all included studies reported all screening performances indexes (such as biopsy rate, proportions of invasive cancers, and proportions of node-negative invasive cancers). Therefore, meta-analysis results from studies reporting screening performances indexes would lead to biased results and complete reporting screening performances for US and MAM screening studies are needed to improve the current results. Fourth, combination data from repeated (longitudinal) US screening for a woman with data from an initial screening would also lead to biased results. Fifth, meta-analyses under the criteria of *P* < 0.05 would potentially overestimate the performations of US even though random-effect model was used. More real-world studies with large sample size are needed in the future.

## Conclusions

Current evidence suggests that S-US screening could detect occult breast cancers missed by MAM. P-US screening has shown to be comparable to P-MAM screening in women with dense breasts in terms of sensitivity, specificity, cancer detection rate, and biopsy rate, but with higher recall rates and higher detection rates for invasive cancers. More studies are needed to investigate the long-term benefits and potential risks (including false positivity, “unnecessary” recalls, and overdiagnosis) of P-US screening. Moreover, we hope that US screening for breast cancer should deserve more attention in the future, not only because US is comparable to MAM in women with dense breasts in terms of sensitivity, specificity, cancer detection rate, and biopsy rate, but also because ultrasound is not a radiation modality and is easier to access in low-resources areas, such as Chinese rural areas.

## Supplementary information

**Additional file 1: Supplementary S1.** Searching strategies in details from four databases. **Supplementary S2.** Flowchart of searching strategy. **Supplementary S3.** Bias risk assessment criteria. **Supplementary S4.** Screening accuracy for S-US screening. **Supplementary S5.** Screening accuracy for P-MAM screening. **Supplementary S6.** Screening accuracy for P-US screening. **Supplementary S7.** Subgroup analyses on the performance of S-US screening for breast cancer. **Supplementary S8.** Subgroup analyses on the performance differences between P-MAM and P-US for breast cancer.

## Data Availability

All data generated or analysed during this study are included in this published article and its supplementary information files.

## Abbreviations

ABUS: Automated whole breast ultrasonography
BR: Biopsy rate
CDR: Cancer detected rate
HHUS: Hand-held ultrasonography
MAM: Mammography
P-US: Primary ultrasound
P-MAM: Primary mammography
ProIC: Proportions of invasive cancers
ProNNIC: Node-negative invasive cancers
RR: Recall rate
S-US: Supplemental ultrasound
US: Ultrasonography
USPSTF: The U.S. preventive services task force
EUSOBI: European society of breast imaging
CACA: Chinese anti-cancer association
