# Supplementary material for: Performance of ultrasonography screening for breast cancer: a systematic review and meta-analysis
Source: BMC Cancer. 2020 Jun 1;20:499. doi: 10.1186/s12885-020-06992-1 (PMC7268243; doi:10.1186/s12885-020-06992-1)
Supplement: Supplementary file 1 — Additional file 1: Supplementary S1. Searching strategies in details from four databases. Supplementary S2. Flowchart of searching strategy. Supplementary S3. Bias risk assessment criteria. Supplementary S4. Screening accuracy for S-US screening. Supplementary S5. Screening accuracy for P-MAM screening. Supplementary S6. Screening accuracy for P-US screening. Supplementary S7. Subgroup analyses on the performance of S-US screening for breast cancer. Supplementary S8. Subgroup analyses on the performance differences between P-MAM and P-US for breast cancer. [file 12885_2020_6992_MOESM1_ESM.docx]

**Supplementary materials**

**Supplementary S1. Searching strategies in details from four databases**

**Pubmed**

| #1 | ((breast neoplasm) OR breast cancer) OR breast carcinoma |
| --- | --- |
| *#2* | (ultrasound) OR ultrasonography |
| #3 | screening |
| *#4* | ((((((((supplemental) OR supplementary) OR adjunct) OR adjunctive) OR combined) OR joint) OR primary) OR single) OR alone |
| #5 | ((((sensitivity) OR specificity) OR detection rate) OR recall rate) OR biopsy rate |
| #6 | ((((#1) AND #2) AND #3) AND #4) AND #5 Filters: Clinical Study; Clinical Trial; Comparative Study; Controlled Clinical Trial; English Abstract; Evaluation Studies; Multicenter Study; Observational Study; Randomized Controlled Trial; Publication date from 2003/01/01 to 2017/12/31; Humans; English; Female; Cancer |

**Scopus**

| #1 | TITLE-ABS-KEY ( "breast neoplasm" OR "breast cancer" OR "breast carcinoma" ) AND DOCTYPE ( ar ) AND PUBYEAR > 2002 |
| --- | --- |
| *#2* | TITLE-ABS-KEY ( "ultrasound" OR "ultrasonography" ) AND DOCTYPE ( ar ) AND PUBYEAR > 2002 |
| #3 | TITLE-ABS-KEY ( screening ) AND DOCTYPE ( ar ) AND PUBYEAR > 2002 |
| *#4* | TITLE-ABS-KEY ( "supplemental" OR "supplementary" OR "adjunct" OR "adjunctive" OR "combined" OR "joint" OR "primary" OR "single" OR "alone" ) AND DOCTYPE ( ar ) AND PUBYEAR > 2002 |
| #5 | TITLE-ABS-KEY ( "sensitivity" OR "specificity" OR "detection rate" OR "recall rate" OR "biopsy rate" ) AND DOCTYPE ( ar ) AND PUBYEAR > 2002 |
| #6 | #1 AND #2 AND #3 AND #4 AND #5 |

**Web of Science**

| #1 | TOPIC: (breast cancer) OR TOPIC: (breast neoplasm) OR TOPIC: (breast carcinoma) |
| --- | --- |
| *#2* | TOPIC: (ultrasound) OR TOPIC: (ultrasonography) |
| #3 | TOPIC: (screening) |
| *#4* | TOPIC: (supplemental) OR TOPIC: (supplementary) OR TOPIC: (adjunct) OR TOPIC: (adjunctive) OR TOPIC: (combined) OR TOPIC: (joint) OR TOPIC: (primary) OR TOPIC: (single) OR TOPIC: (alone) |
| #5 | TOPIC: (sensitivity) OR TOPIC: (specificity) OR TOPIC: (detection rate) OR TOPIC: (recall rate) OR TOPIC: (biopsy rate) |
| *#6* | #5 AND #4 AND #3 AND #2 AND #1 |
| #7 | #5 AND #4 AND #3 AND #2 AND #1 Refined by: PUBLICATION YEARS: ( 2016 OR 2005 OR 2003 OR 2015 OR 2008 OR 2013 OR 2009 OR 2010 OR 2012 OR 2011 OR 2006 OR 2017 OR 2007 OR 2014 OR 2004 ) AND DOCUMENT TYPES: ( ARTICLE ) AND LANGUAGES: ( ENGLISH ) |

**Embase**

('breast neoplasm':ti,ab,kw OR 'breast cancer':ti,ab,kw OR 'breast carcinoma':ti,ab,kw) AND

('ultrasound':ti,ab,kw OR 'ultrasonography':ti,ab,kw) AND

'screening':ti,ab,kw AND

('supplemental':ti,ab,kw OR 'supplementary':ti,ab,kw OR 'adjunct':ti,ab,kw OR 'adjunctive':ti,ab,kw OR 'combined':ti,ab,kw OR 'joint':ti,ab,kw OR 'primary':ti,ab,kw OR 'single':ti,ab,kw OR 'alone':ti,ab,kw) AND

('sensitivity':ti,ab,kw OR 'specificity':ti,ab,kw OR 'detection rate':ti,ab,kw OR 'recall rate':ti,ab,kw OR 'biopsy rate':ti,ab,kw) AND

([controlled clinical trial]/lim OR [randomized controlled trial]/lim) AND [2003-2018]/py

**Supplementary S2. Flowchart of searching strategy.**


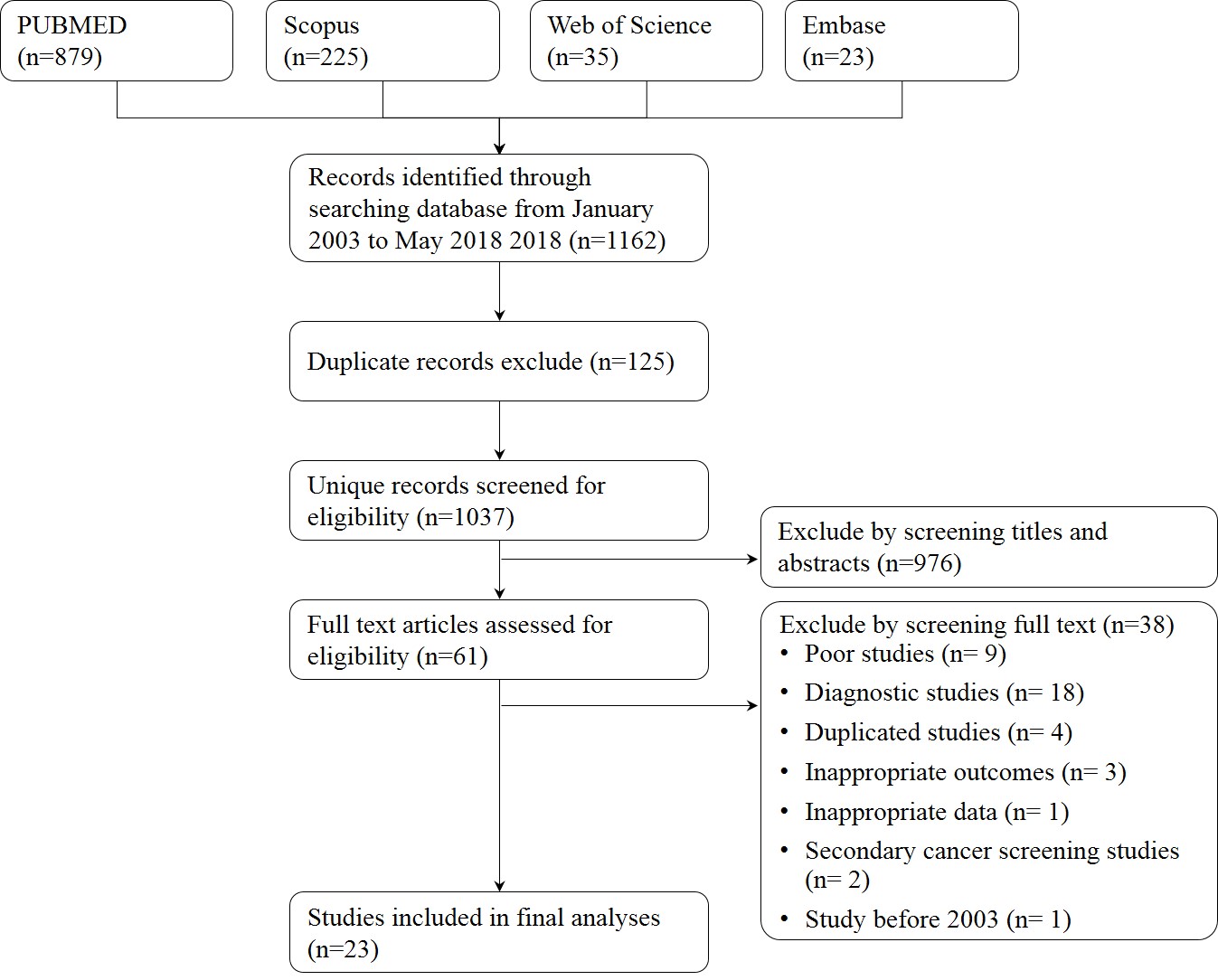


**Supplementary S3. Bias risk assessment criteria**

| **Items** | Low-risk | High-risk |
| --- | --- | --- |
| **Source of**  **population** | General community women or well-defined high-risk women | Women participating in opportunistic screening or other undefined women |
| **Sample size** | Greater than or equal to 1000 | Less than 1000 |
| **Inclusion and exclusion criteria** | Clearly described the inclusion and exclusion criteria, and women who had a personal history of breast cancer were excluded before screening | No clear inclusion and exclusion criteria, or not excluding women who had a personal history of breast cancer before screening |
| **Blinding of**  **tests** | Readers of different screening methods were masked to each other | No blind between different screening methods |
| **Data completeness** | All participants received different methods, or the proportion of missing data for either test was less than or equal to 5%. | The proportion of missing data for either test was greater than 5% |
| **BIRADS criteria** | US findings were interpreted according to BI-RADS criteria | US findings were not interpreted according to BI-RADS criteria |
| **Reference standards** | Women with positive results from index screening methods were ascertained with histopathology; and women with negative results were ascertained with a minimum 12-month clinical follow-up | The case report of women with positive screening results comes from cancer registration or death registration; clinical follow-up for women with negative screening results is less than 12 months |

**Supplementary S4. Screening accuracy for S-US screening**


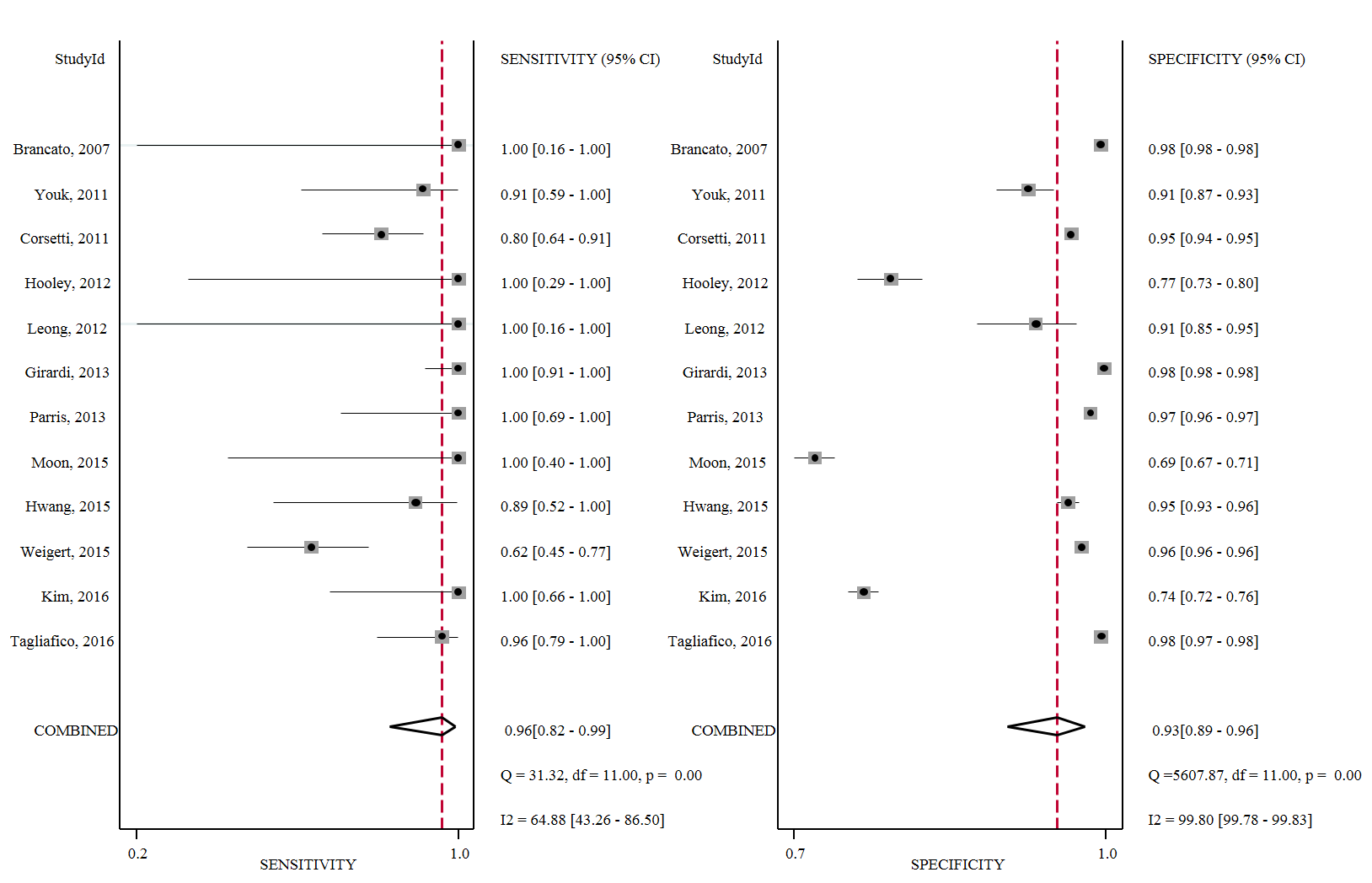


**Supplementary S5. Screening accuracy for P-MAM screening**


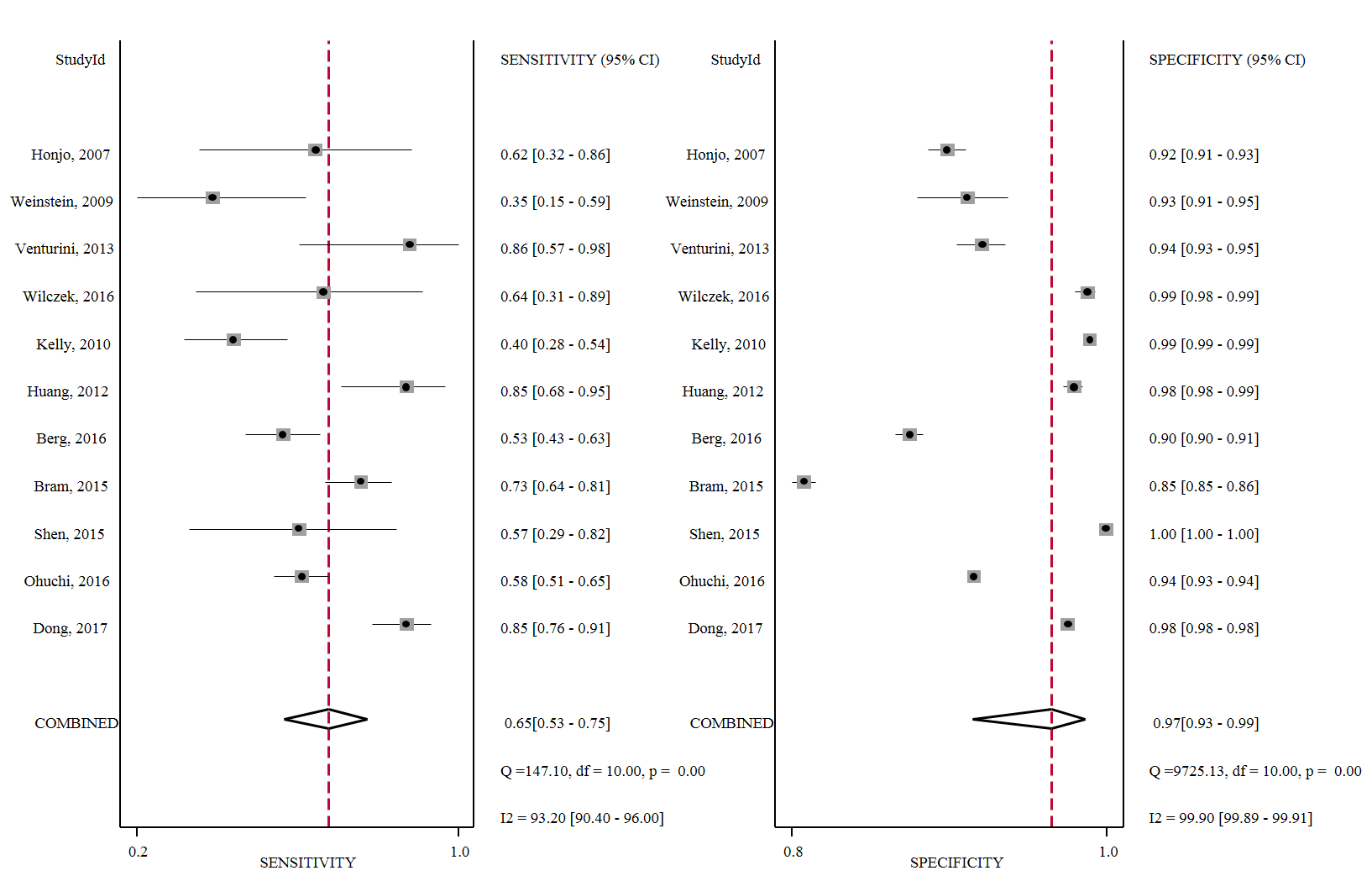


**Supplementary S6. Screening accuracy for P-US screening**


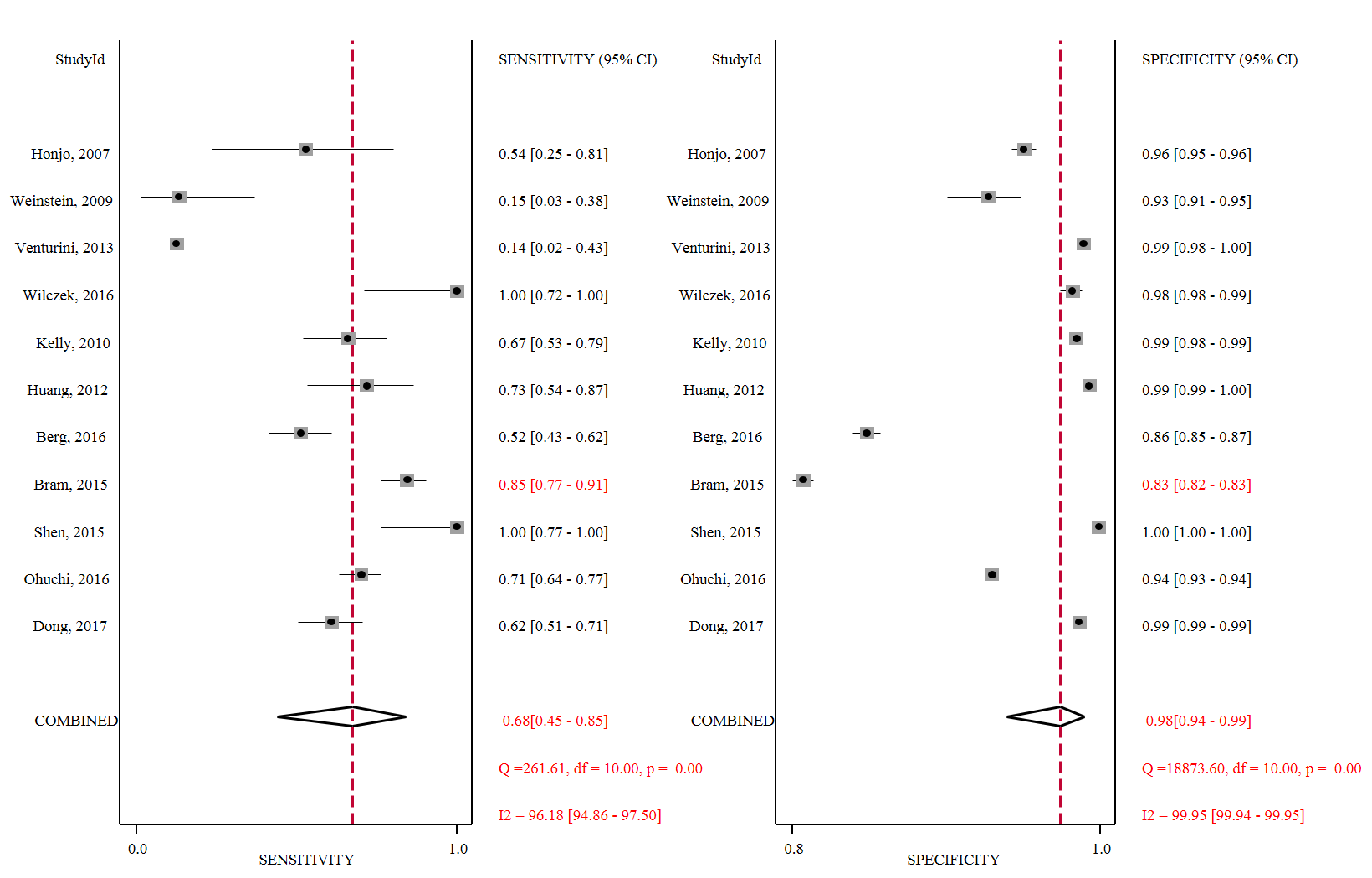


**Supplementary S7. Subgroup analyses on the performance of S-US screening for breast cancer**

| Subgroup | Sensitivity, % | |  | Specificity, % | |  | Cancer detected rate,  1/1000 | |  | Recall rate, % | |  | Biopsy rate, % | |  | ProIC, % | |  | ProNNIC, % | |
| --- | --- | --- | --- | --- | --- | --- | --- | --- | --- | --- | --- | --- | --- | --- | --- | --- | --- | --- | --- | --- |
|  | N | 95%CI |  | N | 95%CI |  | N | 95%CI |  | N | 95%CI |  | N | 95%CI |  | N | 95%CI |  | N | 95%CI |
| All women with dense breasts |  |  |  |  |  |  |  |  |  |  |  |  |  |  |  |  |  |  |  |  |
| Yes | 9 | 93.4 (80.9, 100) |  | 9 | 92.3 (87.5, 96.0) |  | 9 | 3.5 (1.1, 5.9) |  | 9 | 8.3 (4.5, 13.2) |  | 8 | 4.6 (2.9, 6.8) |  | 5 | 73.4 (40.2, 97.6) |  | 2 | 72.0 (47.2, 92.4) |
| No | 3 | 99.9 (89.2, 100) |  | 3 | 90.2 (68.7, 99.8) |  | 3 | 2.4 (1.1, 4.1) |  | 3 | 10.2 (0.3, 31.5) |  | 2 | 1.9 (1.7, 2.1) |  | 2 | 77.2 (46.5, 98.4) |  | 1 | 50.0 (1.3, 98.7) |
| Sample size |  |  |  |  |  |  |  |  |  |  |  |  |  |  |  |  |  |  |  |  |
| ≥ 1000 | 9 | 94.9 (81.9, 100) |  | 9 | 93.2 (88.3, 96.8) |  | 9 | 2.6 (1.6, 3.9) |  | 9 | 7.2 (3.5, 12.1) |  | 7 | 2.7 (1.6, 4.0) |  | 5 | 75.3 (47.3, 96.0) |  | 2 | 65.9 (40.7, 87.9) |
| < 1000 | 3 | 97.8 (76.9, 100) |  | 3 | 85.6 (74.4, 94.0) |  | 3 | 11.9 (1.9, 28.5) |  | 3 | 14.7 (7.0, 24.7) |  | 3 | 9.0 (6.4, 12.1) |  | 2 | 60.3 (10.3, 99.7) |  | 1 | 0.0 (0.0, 84.2) |
| Screening mode |  |  |  |  |  |  |  |  |  |  |  |  |  |  |  |  |  |  |  |  |
| Opportunistic screening | 10 | 95.0 (82.0, 100) |  | 10 | 91.0 (85.6, 95.2) |  | 10 | 2.7 (1.6, 4.1) |  | 10 | 9.5 (5.1, 14.9) |  | 8 | 4.0 (2.6, 5.7) |  | 5 | 64.7 (44.4, 83.1) |  | 2 | 65.9 (40.7, 87.9) |
| Community women | 2 | 100 (89.0, 100) |  | 2 | 98.0 (97.5, 98.5) |  | 2 | 5.9 (3.3, 9.1) |  | 2 | 2.8 (2.2, 33.9) |  | 2 | 1.5 (1.1, 1.9) |  | 2 | 98.5 (83.9, 100) |  | 1 | 100.0 (15.8, 100) |
| Exclusion of BC |  |  |  |  |  |  |  |  |  |  |  |  |  |  |  |  |  |  |  |  |
| Yes | 3 | 92.2 (77.4, 100) |  | 3 | 91.2 (76.0, 99.2) |  | 3 | 4.6 (2.7, 7.0) |  | 3 | 9.5 (1.2, 24.3) |  | 3 | 3.6 (1.5, 6.6) |  | 2 | 93.4 (79.2, 99.8) |  | 1 | 65.0 (40.8, 84.6) |
| No or NR | 9 | 96.8 (80.5, 100) |  | 9 | 92.0 (87.3, 95.7) |  | 9 | 2.4 (1.1, 4.0) |  | 9 | 8.6 (4.7, 13.4) |  | 7 | 4.0 (2.7,5.7) |  | 5 | 58.9 (38.5, 78.1) |  | 2 | 82.0 (22.8, 100.0) |
| Complete data |  |  |  |  |  |  |  |  |  |  |  |  |  |  |  |  |  |  |  |  |
| Yes | 11 | 96.5 (85.1, 100) |  | 11 | 91.5 (86.5, 95.4) |  | 11 | 2.9 (1.6, 4.5) |  | 11 | 9.1 (5.0, 14.1) |  | 9 | 4.2 (2.8, 5.9) |  | 6 | 70.6 (40.8, 94.2) |  | 3 | 70.9 (46.0, 91.6) |
| No | 1 | 88.9 (51.8, 99.7) |  | 1 | 94.6 (94.5, 95.7) |  | 1 | 4.6 (2.0, 9.1) |  | 1 | 5.8 (4.7, 7.0) |  | 1 | 2.1 (1.5, 2.9) |  | 1 | 87.5 (47.4, 99.7) |  | 0 |  |
| BIRADS criteria |  |  |  |  |  |  |  |  |  |  |  |  |  |  |  |  |  |  |  |  |
| Yes | 8 | 95.0 (82.1, 100) |  | 8 | 92.0 (87.1, 96.0) |  | 8 | 4.2 (2.1, 6.8) |  | 8 | 8.4 (4.4, 13.5) |  | 6 | 3.6 (1.3, 5.1) |  | 4 | 61.0 (36.6, 83.3) |  | 2 | 82.0 (22.8, 100.0) |
| No | 4 | 96.0 (78.5, 100) |  | 4 | 91.2 (78.4, 98.6) |  | 4 | 2.4 (1.3, 4.0) |  | 4 | 9.6 (1.9, 22.1) |  | 4 | 4.5 (2.2, 7.5) |  | 3 | 88.9 (60.3, 100) |  | 1 | 65.0 (40.8, 84.6) |
| Follow-up |  |  |  |  |  |  |  |  |  |  |  |  |  |  |  |  |  |  |  |  |
| ≥ 12 months | 7 | 92.0 (82.6, 98.6) |  | 7 | 85.8 (74.8, 94.0) |  | 7 | 4.5 (2.2, 7.4) |  | 7 | 15.0 (6.7, 25.8) |  | 6 | 5.9 (4.1, 8.0) |  | 5 | 75.6 (53.8, 93.1) |  | 2 | 82.0 (22.8, 100.0) |
| <12 months or NR | 5 | 96.2 (73.3, 100) |  | 5 | 97.5 (96.4, 98.4) |  | 5 | 2.2 (1.0, 3.8) |  | 5 | 2.8 (1.9, 3.9) |  | 4 | 1.9 (1.2, 2.7) |  | 2 | 75.3 (61.2, 87.2) |  | 1 | 65.0 (40.8, 84.6) |

**Supplementary S8. Subgroup analyses on the performance differences between P-MAM and P-US for breast cancer**

| Subgroup | Sensitivity, % | |  | Specificity, % | |  | Cancer detected rate,  1/1000 | |  | Recall rate, % | |  | Biopsy rate, % | |  | ProIC, % | |  | ProNNIC, % | |
| --- | --- | --- | --- | --- | --- | --- | --- | --- | --- | --- | --- | --- | --- | --- | --- | --- | --- | --- | --- | --- |
|  | N | 95%CI |  | N | 95%CI |  | N | 95%CI |  | N | 95%CI |  | N | 95%CI |  | N | 95%CI |  | N | 95%CI |
| All women with dense breasts |  |  |  |  |  |  |  |  |  |  |  |  |  |  |  |  |  |  |  |  |
| Yes | 3 | -11.2 (-26.4, 4.1) | | 3 | **2.6 (0.5, 4.6)** |  | 3 | -1.0 (-2.0, 1.0) |  | 3 | **-0.9 (-1.9, -0.0)** |  | 3 | -1.7 (-4.5, 1.2) |  | 3 | **-17.8 (-27.2, -8.4)** | | 1 | 1.6(-6.3, 9.5) |
| No or NR | 8 | 6.1 (-14.7, 26.9) | | 8 | **-0.9 (-1.5, -0.4)** | | 8 | -0.0 (-1.0, 1.0) |  | 8 | **-2.7 (-4.4, -0.9)** |  | 4 | -0.4 (-0.9, 0.1) |  | 7 | **-15.6 (-23.2, -8.1)** |  | 4 | -4.0 (-14.7, 6.7) |
| Type of ultrasound |  |  |  |  |  |  |  |  |  |  |  |  |  |  |  |  |  |  |  |  |
| HHUS | 8 | 9.6 (-8.9, 28.0) | | 8 | -0.6 (-1.3, 0.1) | | 8 | 0.0 (-1.0, 1.0) |  | 8 | **-1.4 (-2.4, -0.4)** | | 4 | -1.3 (-3.7, 1.1) |  | 7 | **-17.0 (-24.7, -9.3)** |  | 4 | -4.0 (-14.7, 6.7) |
| ABUS | 3 | **-20.6(-34.4, -6.8)** | | 3 | 1.3 (0.0, 2.7) |  | 3 | -1.0(-3.0, 0.0) |  | 3 | **-1.6 (-2.9, -0.3)** | | 3 | -0.4 (-1.2, 0.3) |  | 3 | **-15.4 (-26.1, -4.6)** | | 1 | 1.6 (-6.3, 9.5) |
| Sample size |  |  |  |  |  |  |  |  |  |  |  |  |  |  |  |  |  |  |  |  |
| ≥ 1000 | 10 | -1.5 (-16.9, 13.9) | | 10 | -0.1 (-0.7, 0.6) |  | 10 | -0.0 (-1.0, 1.0) |  | 10 | -1.5 (-2.5, 3.5) | | 6 | -1.1 (-2.1, 0.0) |  | 9 | **-15.7 (-21.5, -10.0)** |  | 4 | -0.4 (-6.8, 6.1) |
| < 1000 | 1 | 20.0 (-6.1, 46.1) | | 1 | -0.2 (-3.1, 2.8) |  | 1 | 7.0 (-4.0, 18.0) | | 1 | 0.5 (-5.5, 3.5) |  | 1 | -0.0 (-2.2, 2.0) |  | 1 | -57.1 (-17.1, 2.9) |  | 1 | 0.0 (-61.1, 61.1) |
| Screening mode |  |  |  |  |  |  |  |  |  |  |  |  |  |  |  |  |  |  |  |  |
| Community/high-risk screening | 10 | -0.9 (-14.2, 12.3) | | 10 | **-1.0 (-1.5, -0.4)** | | 10 | -0.0(-1.0, 0.0) |  | 10 | **-0.9 (-1.5, -0.2)** |  | 7 | -1.0 (-2.0, 0.1) |  | 9 | **-17.4 (-23.5, -11.2)** |  | 5 | -0.3 (-6.7, 6.0) |
| Opportunistic women | 1 | 12.1 (-7.4, 31.6) | | 1 | 0.0 (-0.5, 0.6) |  | 1 | 1.0 (-3.0, 6.0) |  | 1 | **-7.0 (-8.3, -5.8)** | | 0 |  |  | 1 | -9.9 (-25.3, 5.5) |  | 0 |  |
| Exclusion of BC |  |  |  |  |  |  |  |  |  |  |  |  |  |  |  |  |  |  |  |  |
| Yes | 8 | 0.9 (-16.4, 18.2) | | 8 | 0.2 (-0.5, 0.9) |  | 8 | -0.0(-1.0, 1.0) |  | 8 | **-2.2 (-3.2, -1.2)** | | 5 | -1.1 (-2.4, 0.3) |  | 7 | **-14.9 (-21.0, -8.8)** |  | 3 | -0.2 (-6.7, 6.3) |
| No or NR | 3 | -1.3(-33.7, 31.2) | | 3 | -1.0 (-3.9, 2.0) |  | 3 | -1.0 (-4.0, 3.0) |  | 3 | 0.9 (-2.2, 4.1) |  | 2 | **-0.8 (-1.4, -0.3)** | | 3 | **-29.7 (-50.4, -9.0)** |  | 2 | -3.0 (-34.6, 28.6) |
| Blinding |  |  |  |  |  |  |  |  |  |  |  |  |  |  |  |  |  |  |  |  |
| Yes | 8 | -2.2 (-17.1, 12.8) | | 8 | -0.0 (-0.6, 0.5) |  | 8 | -0.0 (-1.0, 1.0) |  | 8 | **-1.0 (-1.9, -0.0)** |  | 4 | -1.5 (-3.5, 0.6) |  | 7 | **-16.7 (-23.6, -9.8)** |  | 4 | -4.0 (-14.7, 6.7) |
| No | 3 | 7.7 (-47.2, 62.7) | | 3 | -0.5 (-4.2, 3.3) |  | 3 | 0.0 (-3.0, 4.0) |  | 3 | **-3.0 (-5.1, -0.8)** | | 3 | -0.2 (-0.9, 0.4) |  | 3 | **-15.4 (-27.8, -3.0)** | | 1 | 1.6 (-6.3, 9.5) |
| Complete data |  |  |  |  |  |  |  |  |  |  |  |  |  |  |  |  |  |  |  |  |
| Yes | 8 | -4.8 (-17.6, 8.1) | | 8 | 0.4 (-0.5, 1.3) |  | 8 | -0.0 (-1.0, 1.0) |  | 8 | **-1.5 (-2.8, -0.1)** |  | 4 | -1.5 (-3.4, 0.5) |  | 8 | **-15.5 (-21.3, -9.7)** |  | 4 | -0.4 (-6.8, 6.1) |
| No | 3 | 16.3 (-48.3, 80.8) | | 3 | -1.8 (-5.5, 2.0) |  | 3 | 2.0 (-3.0, 7.0) |  | 3 | -1.8 (-5.5, 1.9) |  | 3 | **-0.1 (-0.3, -0.0)** | | 2 | **-45.8 (-80.0, -11.6)** |  | 1 | 0.0 (-61.0, 61.1) |
| BIRADS criteria |  |  |  |  |  |  |  |  |  |  |  |  |  |  |  |  |  |  |  |  |
| Yes | 8 | 0.1 (-16.1, 16.4) | | 8 | 0.3 (-0.6, 1.1) |  | 8 | -1.0 (-1.0, 0.0) |  | 8 | **-2.5 (-3.7, -1.3)** | | 6 | -1.0 (-2.2, 0.2) |  | 7 | **-16.9 (-23.0, -10.7)** | | 3 | -0.3 (-6.9, 6.3) |
| No | 3 | -0.6 (-39.2, 38.0) | | 3 | -1.0 (-2.5, 0.5) |  | 3 | 1.0 (-0.0, 1.0) |  | 3 | 1.0 (-0.7, 2.7) |  | 1 | -**0.7 (-1.4, -0.0)** |  | 3 | -13.7 (-30.8, 3.3) |  | 2 | -0.6 (-23.2, 22.1) |
| Follow-up |  |  |  |  |  |  |  |  |  |  |  |  |  |  |  |  |  |  |  |  |
| ≥ 12 months | 10 | -5.9 (-18.4, 6.5) | | 10 | 0.3 (-0.2, 0.9) |  | 10 | -0.0 (-1.0, 0.0) |  | 10 | **-1.2 (-2.0, -0.3)** | | 6 | -1.1 (-2.2, 0.1) |  | 9 | **-16.7 (-21.9, -10.3)** |  | 5 | -0.3 (-6.7, 6.0) |
| <12 months or NR | 1 | **71.4 (45.5, 97.4)** | | 1 | **-5.0 (-6.3, -3.7)** | | 1 | 5.0 (-0.0, 10.0) | | 1 | **-5.9 (-8.2, -3.6)** | | 1 | -0.4 (-1.2, 0.5) |  | 1 | -33.3 (-3.7, 37.0) |  | 0 |  |
| Quality |  |  |  |  |  |  |  |  |  |  |  |  |  |  |  |  |  |  |  |  |
| Good | 7 | -6.8 (-20.8, 7.2) | | 7 | **0.7 (0.1, 1.3)** |  | 7 | -0.0 (-1.0, 0.0) |  | 7 | **-1.9 (-2.8, -0.9)** | | 4 | -1.3 (-2.8, 0.2) |  | 6 | **-15.0 (-20.9, -9.1)** |  | 3 | -0.2 (-6.7, 6.3) |
| Fair | 4 | 16.2 (-29.7, 62.1) | | 4 | -2.0 (-5.0, 1.0) |  | 4 | 1.0 (-2.0, 4.0) |  | 4 | -0.7 (-4.1, 2.7) |  | 3 | **-0.5 (-1.1, -0.0)** |  | 4 | **-33.3 (-54.4, -12.1)** | | 2 | -3.0 (-34.6, 28.6) |
